# Research progress on the use of traditional Chinese medicine to treat diseases by regulating ferroptosis

**DOI:** 10.1016/j.gendis.2024.101451

**Published:** 2024-11-06

**Authors:** Shuai Liu, Xianzhen Yang, Sanxia Zheng, Changjing Chen, Lei Qi, Xiangdong Xu, Denglu Zhang

**Affiliations:** a Central Laboratory, Affiliated Hospital of Shandong University of Traditional Chinese Medicine, Jinan, Shandong 250014, China; b Shandong Key Laboratory of Dominant Diseases of Traditional Chinese Medicine, Affiliated Hospital of Shandong University of Traditional Chinese Medicine, Jinan, Shandong 250014, China; c Urinary Surgery, Affiliated Hospital of Shandong University of Traditional Chinese Medicine, Jinan, Shandong 250014, China; d Second Affiliated Hospital of Shandong University of Traditional Chinese Medicine, Jinan, Shandong 250014, China

**Keywords:** Chemical construction, Compounds, Ferroptosis, Monomers, Traditional Chinese medicine

## Abstract

Ferroptosis is an emerging form of programmed cell death triggered by iron-dependent lipid peroxidation. It is distinguished from other forms of cell death by its unique morphological changes and characteristic fine-tuned regulatory gene network. Since its pivotal involvement in the pathogenesis and therapeutic interventions of various diseases, such as malignant tumors, cardiovascular and cerebrovascular diseases, and traumatic disorders, has been well-established, ferroptosis has garnered significant attention in contemporary physiological and pathological research. For the advantage of alleviating the clinical symptoms and improving life quality, traditional Chinese medicine (TCM) holds a significant position in the treatment of these ailments. Moreover, increasing studies revealed that TCM compounds and monomers showed evident therapeutic efficacy by regulating ferroptosis via signaling pathways that tightly regulate redox reactions, iron ion homeostasis, lipid peroxidation, and glutathione metabolism. In this paper, we summarized the current knowledge of TCM compounds and monomers in regulating ferroptosis, aiming to provide a comprehensive review of disease management by TCM decoction, Chinese patent medicine, and natural products deriving from TCM through ferroptosis modulation. The formulation composition, chemical structure, and possible targets or mechanisms presented here offer valuable insights into the advancement of TCM exploration.

## Introduction

In 2012, Dixon et al^1^ proposed the concept of ferroptosis. Ferroptosis is a complex process involving the antioxidant system (SLC7A11, GPX4, FSP1, NADPH, *etc*.), iron metabolism (TFRC, STEAP3, FTH, FTL1, *etc*.), and lipid metabolism (ACSL4, LPCAT3, ALOXs, *etc*.).^2^ The regulation of the above key molecules potentially influences the occurrence of ferroptosis. Ferroptosis is a type of cell death that is dependent on reactive oxygen species (ROS) and is characterized by the accumulation of iron and lipid peroxidation. This process is closely linked to the reductive/oxidative (redox) metabolic processes that occur within cells.

The ferroptosis process has been implicated in many diseases, such as cardiovascular disease, ischemia-reperfusion injury, neurodegenerative disease, and cancer.^3^, ^4^, ^5^, ^6^ Traditional Chinese medicine (TCM) has been used extensively in the clinic to treat the above diseases and demonstrates evident therapeutic efficacy. In recent years, there has been a proliferation of studies highlighting the therapeutic abilities of TCM, encompassing both compounds and monomers, to modulate ferroptosis and thereby achieve disease treatment. TCM possesses distinct advantages in regulating redox-dependent metabolic processes, as evidenced by multiple studies demonstrating its efficacy in mitigating disorders through the modulation of ferroptosis.^7^ The therapeutic foundation of TCM is based on its active pharmaceutical constituents, which can be categorized based on their chemical composition. These include glycosides, quinoids, flavonoids, terpenoids, organic acids, alkaloids, and other compounds. It is expected that the integration of TCM, natural medicinal chemistry technology, and contemporary pharmacological experimental methods will facilitate the investigation of the correlation between the structure and activity of the active ingredients. Additionally, this approach will enable the identification of key compounds and facilitate structural modification and transformation. Ultimately, this research will serve as a foundation for the development of novel medications characterized by enhanced efficacy, improved target selectivity, reduced toxicity and side effects, and enhanced safety.

## Characteristics of ferroptosis

**Figure 1:**
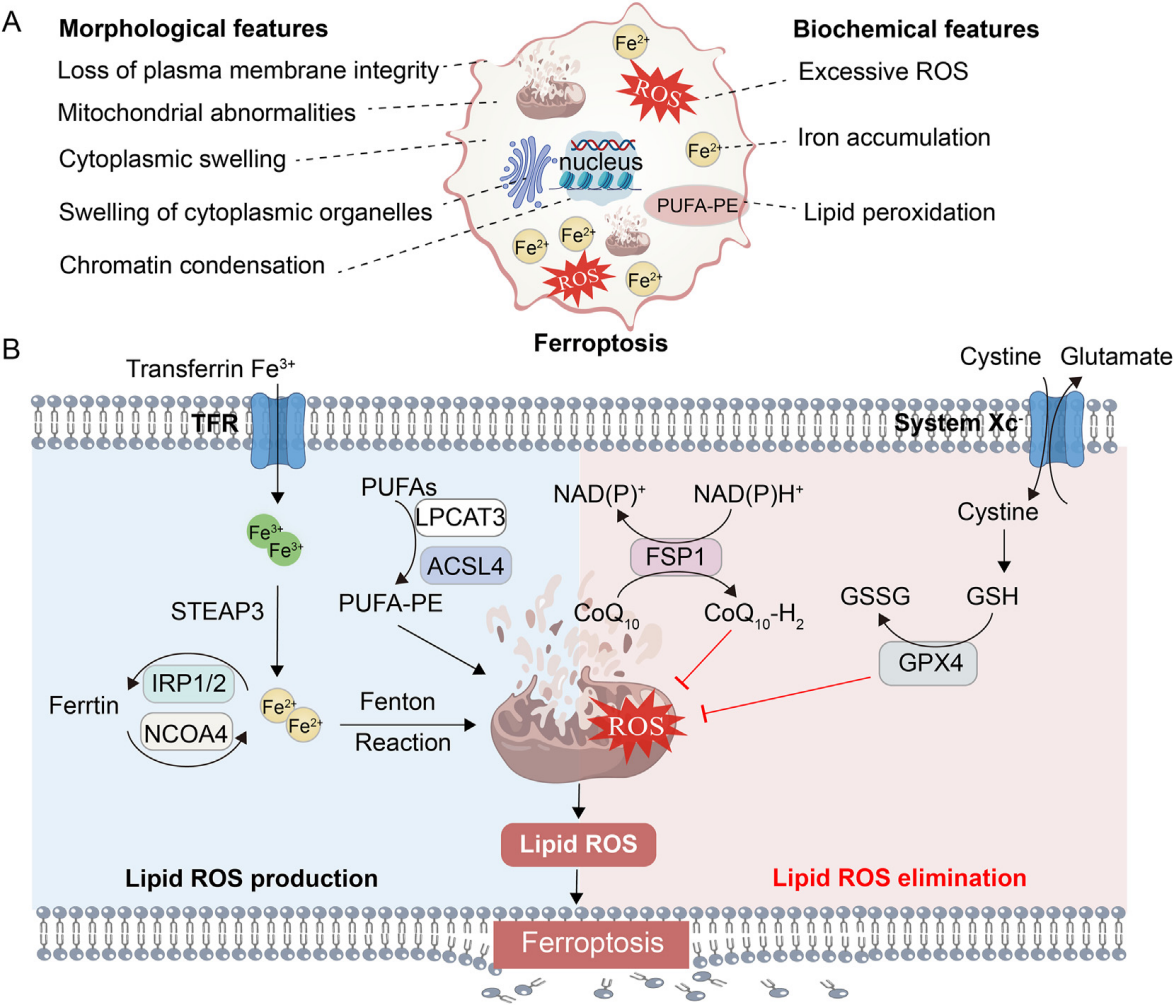
Characteristics of ferroptosis. **(A)** Morphological and biochemical characteristics of ferroptosis. **(B)** Core regulatory proteins of ferroptosis.

Ferroptosis is a novel form of programmed cell death characterized by iron-dependent lipid peroxidation. This type of death is distinct from traditional forms such as apoptosis and necrosis. Morphologically, cells undergoing ferroptosis exhibit necrosis-like changes, including loss of membrane integrity, cytoplasmic swelling, intracellular organelle swelling, and moderate chromatin condensation.^2^ Ferroptosis causes ultrastructural abnormalities in mitochondria, including reduced or absent cristae, rupture of the outer membrane, or increased membrane density^2^ (Fig. 1A).

The process of ferroptosis has distinct biochemical characteristics, such as excessive ROS, iron accumulation, and lipid peroxidation (Fig. 1A). During ferroptosis, there is either overexpression or low expression of key proteins. The abnormal expression of these proteins is closely related to the biochemical characteristics of ferroptosis (Fig. 1B). Excessive ROS is mainly caused by an imbalance in the redox state. The proteins regulating redox state in cells are primarily anti-oxidant system proteins, including GPX4 (glutathione peroxidase 4), NRF2 (nuclear factor erythroid 2-related factor 2), SLC7A11 (solute carrier family 7 member 11), and NADPH (nicotinamide adenine dinucleotide phosphate).^8^ The proteins related to iron accumulation include TFRC (transferrin receptor), STEAP3 (six-transmembrane epithelial antigen of prostate 3), FTH (ferritin heavy chain), and FTL1 (ferritin light chain 1).^2^ In ferroptosis, specific membrane lipid peroxidation plays a decisive role in cell death, with ACSL4 (acyl-CoA synthetase long-chain family member 4) and LPCAT3 (lysophosphatidylcholine acyltransferase 3) playing key roles in this process.^9^ The proteins mentioned above are crucial in ferroptosis and become key targets for its regulation.

## TCM compounds regulating ferroptosis

Malignant tumors, cardiovascular diseases, cerebrovascular diseases, and traumatic diseases are significant maladies that pose a threat to human health. Ferroptosis plays a pivotal role in the pathogenesis and progression of these diseases. TCM compounds have greatly contributed to the therapeutic management of these diseases. Consequently, the classification of disorders is one method that can be used to assess the relationship between the control of ferroptosis and these compounds.

## Malignant tumors

*Fuzheng Kang’ai Decoction*, a formulation comprising 12 Chinese herbal medicines, namely, *Radix Pseudostellariae*, *Rhizoma Atractylodis macrocephalae*, *Milkvetch Root*, *Hedyotis Diffusa*, *Solanum Nigrum*, *Chinese Sage Herb*, *Indian Iphigenia Bulb*, *Coix Seed*, *Akebia Trifoliata Koidz*, *Snake Bubble Ilicifolius*, *Curcuma Zedoaria*, and *Licorice*, has been extensively utilized in China for several decades as a treatment for patients with non-small cell lung cancer (NSCLC), and it exhibits unequivocal therapeutic outcomes in clinical settings.^10^, ^11^, ^12^ Zhao et al^13^ discovered that *Fuzheng Kang’ai Decoction* effectively triggered ferroptosis in NSCLC cells through the augmentation of lipid peroxidation and the abundance of intracellular Fe^2+^ ions. Subsequent investigations have substantiated the pivotal involvement of GPX4 in the induction of *Fuzheng Kang’ai Decoction*-mediated ferroptosis in NSCLC cells.^13^

*Qingre Huoxue Formula*, comprising *Scutellaria baicalensis* and *Radix paeoniae rubra*, is frequently used in the treatment of chronic obstructive pulmonary disease and lung cancer.^14^ Xu et al^14^ discovered that *Qingre Huoxue Formula* exhibits the ability to stimulate ferroptosis and apoptosis, thereby inhibiting the growth of NSCLC cells through the involvement of the p53 and GSK-3β (glycogen synthase kinase 3 beta)/Nrf2 signaling pathways.

*Fuzheng Nizeng Decoction*, a derivative of the renowned *Liujunzi Decoction*, has demonstrated favorable clinical efficacy in the treatment of gastric precancerous lesions according to clinical studies.^15^ Chu et al^16^ discovered that *Fuzheng Nizeng Decoction* can induce ferroptosis and endoplasmic reticulum stress in MNNG (N-methyl-N′’-nitro-N-nitrosoguanidine)-induced gastric precancerous lesion cells, leading to a reduction in GPX4/glutathione (GSH) levels. Furthermore, it was observed that the ATF3 (activating transcription factor 3)/CHOP (CCAAT-enhancer-binding protein homologous protein)/CHAC1 (ChaC glutathione specific gamma-glutamylcyclotransferase 1) signaling pathway may engage in crosstalk, thereby offering a novel molecular mechanism for the management of gastric precancerous lesions.^16^

The formulation of *Shuganning Injection* is derived from the traditional prescription *Yinchenhao Decoction*, which was originally documented in the *Treatise on Cold Damage Diseases* compiled by Zhongjing Zhang approximately 1800 years ago. *Shuganning Injection* received approval as a traditional Chinese patent medicine from the China Food and Drug Administration in 2002, as stated in the Pharmacopoeia of 2005. Due to its hepatoprotective and immunomodulatory properties, *Shuganning Injection* is extensively utilized as an adjuvant therapy for cancer, particularly in cases of hepatic carcinomas.^17^^,^^18^ Du et al^19^ discovered that the induction of ferroptosis by *Shuganning Injection* was contingent upon the presence of HO-1 (heme oxygenase 1), which facilitated the accumulation of labile iron pools within the cell. This effect was mitigated through the knockdown of HO-1 and the inhibition of its activity using tin protoporphyrin IX.^19^

## Cardiovascular and cerebrovascular diseases

The composition of *DiDang Decoction* (DDD) includes rhubarb, peach seed, leech, and gadfly. It is known for its ability to alleviate stagnancy and eliminate blood stasis, as originally documented in the *Treatise on Febrile Diseases and Synopsis of Prescriptions of the Golden Chamber*. Over several years, it has been extensively utilized in clinical practice for the treatment of various metabolic disorders. Furthermore, numerous randomized control trials have demonstrated the significant efficacy of *DiDang Decoction* in managing atherosclerosis and hyperlipidemia.^20^^,^^21^ Wu et al^22^ provided evidence that DDD exhibited therapeutic effects on atherosclerosis and hyperlipidemia by targeting multiple pathways and mechanisms, leading to improvements in mitochondrial function, reduction in ROS levels, and inhibition of ferroptosis and apoptosis, which were achieved through the activation of the HIF-1 (hypoxia-inducible factor 1) signaling pathway.

Academician Chen Keji’s representative prescription, *Qingxin Jieyu Granule*, is formulated based on the etiology and pathogenesis of “blood stasis pattern” and is commonly used for the treatment of atherosclerotic cardiovascular diseases.^23^ A network pharmacology study conducted by Zhang et al revealed that the regulation of macrophage ferroptosis serves as a crucial pathway through which *Qingxin Jieyu Granule* effectively stabilizes vulnerable atherosclerosis plaques. The study further demonstrated that *Qingxin Jieyu Granule* exerts its inhibitory effects on ferroptosis in vulnerable atherosclerosis plaques, partially mediated by the GPX4/xCT (the cystine/glutamate antiporter SLC7A11) signaling pathway.^24^

*Foshousan*, a traditional Chinese herbal decoction, was first documented in *Puji Benshi Fang* during the Song Dynasty (AD 1132) by Shuwei Xu. It consists of *Angelica sinensis* and *Ligusticum wallichii* in a weight ratio of 3:2.^25^ In recent years, it has been extensively utilized in the treatment of diverse ailments, with a particular focus on cerebral vascular impairment.^26^^,^^27^ Wang et al^25^ conducted a study demonstrating the potential of *Foshousan* to mitigate cognitive deficits induced by chronic cerebral hypoperfusion through modulation of the NRF2/HO-1 pathway against ferroptosis. Additionally, *Compound Tongluo Decoction*, a TCM formulation, exhibits a range of pharmacological abilities, such as hepatic and renal nourishment, phlegm dissipation, and blood stasis removal.^28^ Clinical studies have demonstrated that it exhibits the potential to ameliorate the symptoms of cerebral infarction, safeguarding the neurological function of convalescent patients, stimulating angiogenesis post-cerebral infarction, and reducing the volume of cerebral infarction.^29^^,^^30^ Further investigation has indicated that *Compound Tongluo Decoction* may impede ferroptosis induced by endoplasmic reticulum stress and facilitate angiogenesis through the activation of the Sonic Hedgehog pathway in rats with cerebral infarction.^28^

*Naotaifang*, a compound Chinese herbal medicine, comprises *Radix Astragali*, *Rhizoma chuanxiong*, *Pheretima*, and *Bombyx batryticatus*. Several studies have demonstrated the clinical efficacy of *Naotaifang* in enhancing nervous system function in patients suffering from acute cerebral ischemia.^31^^,^^32^ In their research, Lan et al^33^ observed that acute cerebral ischemia triggers neuronal ferroptosis, and the administration of *Naotaifang* to rats with middle cerebral artery occlusion resulted in the suppression of ferroptosis through activation of the TFR1 (transferrin receptor 1)/DMT1 (divalent metal transporter 1) and SCL7A11/GPX4 pathways.

*Qishen Yiqi Dripping Pill* is a TCM comprising four medicinal herbs, namely, *Astragalus membranaceus Fisch. ex Bunge*, *Salvia miltiorrhiza Bge.*, *Panax notoginseng (Burk.) F. H. Chen*, and *Dalbergia odorifera T. Chen*.^34^ The National Medical Products Administration approved its use in 2003 for the clinical treatment of ischemic heart diseases. In their study, Wu et al^35^ discovered that *Qishen Yiqi Dripping Pill* has the potential to alleviate myocardial ischemia-induced ferroptosis by enhancing mitochondrial biogenesis and dynamic homeostasis.

## Traumatic diseases

*Niujiaodihuang Detoxify Decoction* (NDD), an integrated TCM prescription, has been used as a therapeutic intervention for acute liver failure (ALF). It comprises nine botanical ingredients, namely, *Cornu Bubali*, *Paeonia lactiflora Pall*, *Rehmannia glutinosa*, *Artemisia anomala S. Moore*, *Artemisia capillaris Thunb*, *Gentiana macrophylla Pall*, *Paeoniae Alba Radix*, *Gardenia jasminoides J. Ellis*, and *Glycyrrhiza inflata Batalin*. Ji et al^36^ conducted a study demonstrating its effectiveness in alleviating liver injury induced by lipopolysaccharide. Subsequent investigations have revealed that *Niujiaodihuang Detoxify Decoction* exerts hepatoprotective effects by modulating the reprogramming of GSH metabolism, thereby inhibiting the progression of liver injury.^36^

*Wenqingyin*, derived from the renowned TCM text “*Rejuvenation of All Diseases*”, has been extensively utilized by medical practitioners for the treatment of diverse infectious ailments. Its composition includes *Coptidis Rhizoma*, *Phellodendri Cortex*, *Gardeniae Fructus*, *Scutellaria Radix*, *Rehmanniae Radix Preparata*, *Paeoniae Alba Radix*, *Angelicae Sinensis Radix*, and *Chuanxiong Rhizoma* in equimolar proportions. Xie et al^37^ conducted a study demonstrating the potential of *Wenqingyin* in ameliorating liver injury induced by lipopolysaccharide. Further study has demonstrated that *Wenqingyin* effectively inhibits ferroptosis during the development of lipopolysaccharide-induced liver injury by activating the Nrf2-mediated signaling pathway.^37^

Derived from the TCM formulations of *Dachaihu Decoction* and *Dachengqi Decoction* in the *Treatise on Cold Damage Diseases* compiled by Zhongjing Zhang, *Qingyi Decoction* (QYD) is frequently used in the treatment of individuals suffering from acute pancreatitis due to its purgative properties and ability to eliminate fever and toxic substances.^38^ In this study, its active ingredients were examined using network pharmacology to identify its primary molecular targets and targeted signaling pathways in the context of severe acute pancreatitis-associated acute lung injury. Rescue experiments were conducted to establish the association between *Qingyi Decoction* and ferroptosis. The findings of this study suggest that *Qingyi Decoction* has the potential to inhibit ferroptosis by enhancing ALDH2 (aldehyde dehydrogenase 2) expression and to mitigate neutrophil infiltration by inhibiting the cleavage of intact AnxA1 (annexin A1) and by down-regulating ICAM1 (intercellular adhesion molecule 1) expression.^39^

*Xiaojianzhong Decoction*, a TCM compound comprising cassia twig, *Paeonia lactiflora*, liquorice, ginger, jujube, and maltose, has been widely recognized for its therapeutic effects on stomach diseases for over two millennia. A recent study demonstrated its significant efficacy in mitigating aspirin-induced gastric mucosal injury and suppressing oxidative stress and ferroptosis in mice.^40^ Subsequent investigations have suggested that the mechanism underlying this protective effect involves the inhibition of aspirin-induced oxidative stress and ferroptosis through the p62/Keap1 (Kelch-like ECH-related protein 1)/Nrf2 signaling pathway, ultimately leading to the amelioration of gastric mucosal injury.^40^

## TCM monomers that can regulate ferroptosis

**Figure 2:**
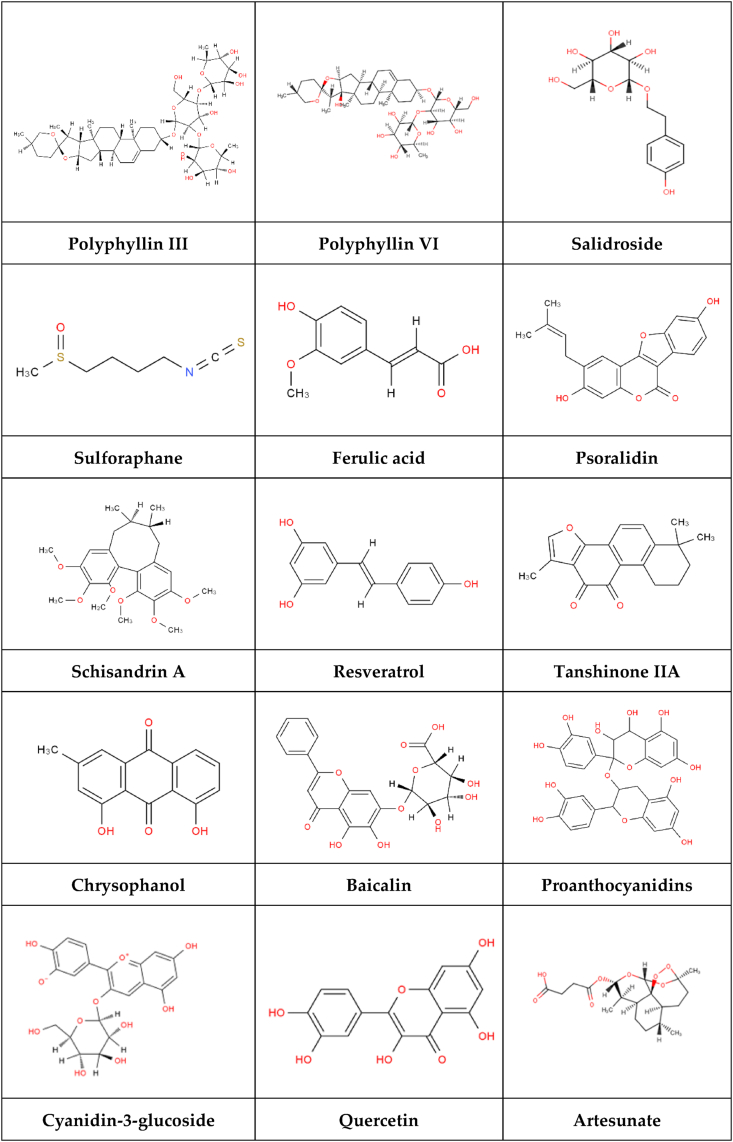

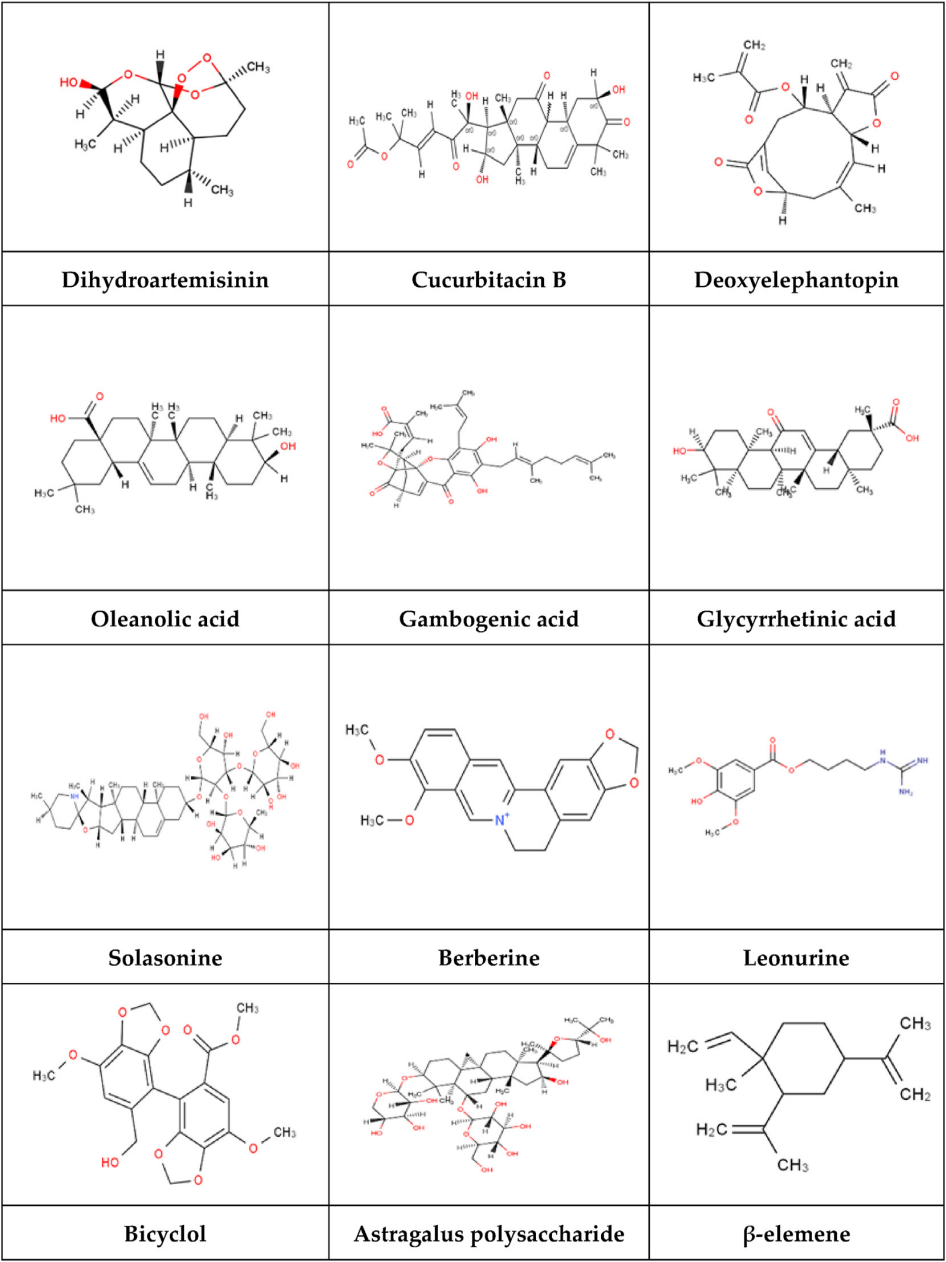
Chemical structure of the monomer.

The material basis of TCM treatment of diseases is its active pharmaceutical components. The chemical structure of a component plays a significant role in determining its physical and chemical properties, as well as its absorption, distribution, metabolism, and excretion within the body. Consequently, we classified the active components of TCM, including glycosides, phenylpropanoids, quinoid compounds, flavonoids, terpenoids, organic acids, alkaloids, and other compounds, based on their respective chemical structures. Figure 2 illustrates the chemical structure of the TCM monomers.

## Glycosides

Glycosides, also referred to as ligands, are compounds resulting from the linkage of the terminal carbon atom of a sugar or sugar derivative with another class of non-sugar substances (aglycones, ligands). Glycosides are widely distributed throughout the plant kingdom, particularly in higher-order plants.

### Polyphyllin

*Paris polyphylla* is a highly useful medicinal plant that is primarily found in the Sichuan and Yunnan provinces of China. Saponins are the primary active substances of the plant. Investigations regarding tumors frequently examine saponins such as polyphyllin I (PPI), polyphyllin III (PPIII), and polyphyllin VI (PPVI).^41^ Zhou et al^42^ explored the anti-cancer effects of PPIII on MDA-MB-231 human breast cancer cells both *in vitro* and *in vivo* and revealed that PPIII exerted anti-cancer activities mainly through ACSL4-mediated lipid peroxidation activation and ferroptosis induction. PPVI has demonstrated potent anti-cancer effects in various malignant tumors. A recent study revealed that PPVI effectively inhibited the proliferation, migration, and invasion of hepatocellular carcinoma cells by potentially targeting the STAT3 (signal transducer and activator of transcription 3)/GPX4 axis to induce ferroptosis.^43^

### Salidroside

Salidroside, a p-hydroxyphenyl-β-glucoside compound, is distributed in all parts of *Rhodiola rosea* L. and has various biological activities and a wide spectrum of pharmacological properties. Yang et al^44^ revealed that salidroside plays a neuroprotective role by inhibiting neuronal ferroptosis in mice with Aβ1−42-induced Alzheimer’s disease and in Glu-injured HT22 cells, and its mechanism is related to activation of the NRF2/HO1 signaling pathway. Next, the researchers used SAMP8 (senescence-accelerated mouse prone 8) mice as Alzheimer’s disease models and treated them with salidroside. The administration of salidroside decreased iron deposition, reduced TFR1 and ACSL4 protein expression, and up-regulated SLC7A11 expression. Further experiments suggested that salidroside alleviates cognitive impairment and inhibits neuronal ferroptosis. The underlying mechanisms may involve Nrf2/GPX4 axis activation and a reduction in CD8^+^ T-cell infiltration.^45^

### Sulforaphane

Sulforaphane, a compound found in broccoli and in broccoli sprouts that is transformed from a significant glucosinolate, has been demonstrated to decrease the development of preexisting tumors and to prevent chemically produced malignancies in animal models.^46^ Sulforaphane prevents environmental carcinogens from harming cells and causes growth arrest and/or apoptosis in numerous cancer cell types.^47^ According to Wang et al,^48^ ferroptosis was observed in the hearts of type 2 diabetic mice with diabetic cardiomyopathy. By increasing ferritin and SLC7A11 levels, sulforaphane-activated NRF2 prevented cardiac cell ferroptosis in both advanced glycation end-product-treated engineered cardiac tissues and hearts of diabetic cardiomyopathy mice. Moreover, AMPK (AMP-activated protein kinase) was required for sulforaphane’s inhibitory action on ferroptosis. This evidence shows that sulforaphane inhibits ferroptosis via the AMPK/NRF2 pathways.^48^

## Phenylpropanoids

Phenylpropanoids are a group of natural organic compounds with one or more C6–C3 units in the basic parent nucleus, including simple phenylpropanoids (phenylpropylene, phenylpropanol, phenylpropanal, phenylpropionic acid, *etc*.), coumarins, and lignans. The existence of phenylpropanoids is related to the regulation of plant growth and resistance to disease. It is a highly significant class of natural substances that exhibits a wide variety of biological activities.

### Ferulic acid

Ferulic acid is a common phenolic acid in plant cell walls. It forms a cross-linked component of the cell wall together with lignin and polysaccharide. It is one of the effective components of various TCMs, such as *Ferula*, *Angelica sinensis*, *Chuanxiong*, and cohosh. Ferulic acid is highly effective at scavenging ROS and activating anti-oxidant enzymes, which are crucial in preventing the development of diabetes, cardiovascular disease, and cancer.^49^, ^50^, ^51^ Liu et al^52^ discovered that ferroptosis contributed to rat myocardial ischemia–reperfusion damage. According to their research, ferulic acid reduced ROS overproduction, promoted GSH production, blocked Ptgs2 (prostaglandin-endoperoxide synthase 2) mRNA and Fe^2+^ accumulation, decreased LDH (lactate dehydrogenase) and CK (creatine kinase) activities, attenuated myocardial infarction, and improved ischemia–reperfusion-induced ferroptosis. It also improved oxidative stress and decreased ROS overproduction. The prospect of creating medications containing ferulic acid to prevent cardiac problems is encouraging.^52^

### Psoralidin

One of the main coumarins found in *Psoralea corylifolia* L. seeds, psoralidin, has been utilized as an alternative treatment for numerous illnesses, including depression, inflammatory diseases, and cardiovascular illnesses.^53^ In a study by Yaseen et al,^54^ MeOH fruit extract of *C. corylifolium* was used to isolate psoralidin, which was then further purified by preparative high-performance liquid chromatography. Psoralidin inhibited ferroptosis in HT22 mouse hippocampal cells exposed to erastin. Additionally, a molecular docking assay revealed that psoralidin might bind to two putative ferroptosis targets: 5-LOX (5-lipoxygenase) and the interface of Keap1-Nrf2 interaction. These results suggest that psoralidin may have therapeutic promise in the management of disorders associated with ferroptosis.^54^

### Schisandrin A

Schisandrin A is a lignan extracted from the dried fruits of *Schisandra chinensis*, a traditional Chinese functional food. It has a variety of pharmacological effects on immune control, apoptosis inhibition, and anti-oxidation.^55^, ^56^, ^57^, ^58^ Wang et al^59^ established *in vitro* and *in vivo* models: streptozotocin was administered to C57BL/6 mice following a high-fat diet to induce diabetes. d-glucose (20 mmol/L) was used to activate human renal glomerular endothelial cells to construct a diabetic nephropathy model. They investigated the function of schisandrin A in both models and discovered that it attenuated ferroptosis in diabetic nephropathy via AdipoR1/AMPK-ROS/mitochondrial damage.^59^

## Quinoids

Quinoid compounds are natural organic compounds that have an unsaturated cyclodione structure (quinoid structure) within the molecule or are easily transformed into such a structure. Quinones are the active constituents in many natural remedies, including rhubarb, *Polygonum multiflorum*, *Polygonum polygonum*, aloe vera, and salviorrhiza. Quinones are mostly found in the metabolites of *Polygonaceae*, *Rubiaceae*, *Liliaceae*, legumes, and other higher-order plants. Quinones are a promising family of natural medicines with potential anti-oxidant, anti-inflammatory, anti-bacterial, diuretic, and hemostatic effects.

### Resveratrol

Resveratrol mainly exists in veratrum pilosa, knotweed, grape, and other plants, and it has immune regulatory, anti-oxidant, anti-inflammatory, myocardial protective, and other biological properties.^60^, ^61^, ^62^, ^63^ Researchers have found that in oxygen–glucose deprivation/reoxygenation-induced H9c2 cells and ischemia–reperfusion rats, resveratrol reduced ferroptosis, decreased TFR1 expression, and elevated the expression of FTH1 (ferritin heavy chain 1) and GPX4. Additionally, they discovered that resveratrol prevented ferroptosis through the control of USP19 (ubiquitin-specific peptidase 19)/Beclin1 autophagy.^64^

### Tanshinone IIA

Tanshinone IIA is the active ingredient extracted from *Salvia miltiorrhiza*, which has anti-angiogenic, anti-oxidant, anti-inflammatory, immunomodulatory, and anti-cancer properties and is recognized as a traditional medicine in China.^65^, ^66^, ^67^, ^68^, ^69^ Through the detection of spheroid formation, flow cytometry analysis, and the expression of stem cell markers (OCT3/4, ALDH1A1, and CD44), Ni et al^70^ discovered that tanshinone IIA might lower gastric cancer cell stemness. According to further analysis, it increased lipid peroxide levels and lowered GSH levels in gastric cancer cells, both of which are indicators of ferroptosis. Moreover, the expression of a ferroptosis inhibitor (Fer-1) or overexpression of SLC7A11, a crucial ferroptosis inhibitor, abolished the inhibitory effects of tanshinone IIA on gastric cancer cell stemness. Thus, tanshinone IIA partially caused ferroptosis, which decreased the stemness of gastric cancer cells.^70^

### Chrysophanol

The primary anthraquinone in rhubarb, chrysophanol, prevents myocardial ischemia and improves lipid metabolism while also having anti-inflammatory, anti-cancer, and neuroprotective properties.^71^^,^^72^ In two separate oral cancer cell lines, FaDu, a hypopharyngeal squamous cell carcinoma cell line, and SAS, a poorly differentiated squamous cell carcinoma cell line from a primary lesion on the human tongue, Lin et al^73^ evaluated the pharmacological effects of chrysophanol on ferroptosis. The findings showed that chrysophanol boosted the levels of lipocalin-2 and CHOP and lowered the levels of GPX4 and lipid ROS. These results imply that chrysophanol has the therapeutic potential to slow the development of oral carcinogenesis by inducing ferroptosis.^73^

## Flavonoids

Flavonoids have a basic skeleton made up of the carbon atoms C6–C3–C6 and consisting of 15 carbon atoms.^74^ Depending on the different substituents on the carbon backbone, flavonoids can be categorized into various subclasses.^75^ They are widespread in nature and typically found in many plant components, particularly in flowers and leaves. Flavonoids have anti-inflammatory, anti-neoplastic, anti-bacterial, anti-oxidant, anti-viral, and other biological actions.^76^, ^77^, ^78^, ^79^, ^80^

### Baicalin

Baicalin (7-glucuronic acid-5,6-dihydroxy-flavone) is a lipophilic flavonoid glycoside isolated from *Scutellaria* root that possesses strong biological activities. When H9c2 cells were treated with oxygen–glucose deprivation/reoxygenation, increased lipid peroxidation, significant iron accumulation, activated TfR1 (transferrin receptor protein 1) signaling, and NCOA4 (nuclear receptor coactivator 4)-mediated ferritinophagy were observed. These effects were all reversed by baicalin treatment, according to research by Fan et al.^81^ Additionally, the protective effect of baicalin produced in H9c2 cells was weakened by the overexpression of ACSL4. They concluded by saying that baicalin protects against myocardial ischemia/reperfusion injury by decreasing ACSL4-controlled ferroptosis.^81^ Moreover, baicalin has been reported to reduce iron deposition in liver, kidney, and brain tissues while improving neural dysfunction by inhibiting ferroptosis in brain tissues.^82^, ^83^, ^84^

### Proanthocyanidins

Natural flavonoids have been demonstrated to be able to stop illnesses caused by lipid peroxidation and inflammation. Safflower (*Carthamus tinctorius* L.) contains flavonoids called proanthocyanidins that have anti-viral, anti-inflammatory, and anti-oxidant properties.^85^^,^^86^ To assess the defense function of proanthocyanidins against influenza A virus-induced acute liver injury and its potential mechanism, Lv et al^87^ used a mouse model of acute liver injury caused by the nasal injection of influenza A virus. When proanthocyanidins are administered, GPX4 and SLC7A11 expression are up-regulated, ACSL4 expression is down-regulated, GSH levels are restored, redox equilibrium is preserved, and anti-oxidative effects are strengthened. Proanthocyanidins can therefore prevent ferroptosis to treat influenza A virus-induced acute liver injury.^87^ Anthocyanins such as cyanidin-3-glucoside belong to a characteristic class of soluble flavonoids. Both *in vitro* and *in vivo*, cyanidin-3-glucoside had an inhibitory effect on ferroptosis, which was characterized by the reduction in excessive intracellular free iron accumulation, a reduction in 4HNE (4-hydroxy-2-nonenal) accumulation, lipid ROS, malondialdehyde levels, and ACSL4 expression, and an increase in GPX4 expression and GSH levels.^88^

### Quercetin

Quercetin has been associated with several advantageous physiological processes, including those with anti-oxidant, anti-diabetic, anti-cancer, and anti-inflammatory benefits.^89^, ^90^, ^91^, ^92^, ^93^ Multiple low-dosage injections of streptozocin were used to create a type 2 diabetes mouse model, which was then treated for four months with quercetin. Research shows that ferroptosis is a factor in pancreatic β cell dysfunction and loss. However, quercetin may reduce pancreatic iron deposition and pancreatic β cell ferroptosis, hence reducing the risk of type 2 diabetes.^94^

## Terpenoids

Terpenoids are chemical substances and their derivatives that are generated from meglutaric acid and have the isoprene unit as their fundamental structural unit. Chinese herbs, fruits, vegetables, and whole grains all contain terpenoids in large quantities. They are a class of metabolites that are abundantly present in plants. Terpenes serve a variety of critical ecological roles in controlling how plants interact with their surroundings. Certain terpenes are essential for plant growth and development, and other terpenes have crucial biological functions.

### Artemisinin and artesunate

Artemisinin is a sesquiterpene lactone compound found in *Artemisia annua* (*Asteraceae*). It was first discovered in 1971 by the Nobel Prize-winning Chinese chemist Youyou Tu. Artemisinin compounds have unique oxygen bridge structures, have significant therapeutic effects on malaria, and do not easily produce drug resistance.^57^ Dihydroartemisinin and artesunate, its derivatives, have both been approved by the US Food and Drug Administration, and it is well acknowledged that they have pharmacological action.^95^, ^96^, ^97^

Artesunate is a water-soluble form of artemisinin. In addition to having potent anti-malarial, anti-parasitic, anti-viral, and other actions, artesunate also has considerable anti-tumor effects.^97^, ^98^, ^99^, ^100^, ^101^, ^102^ *In vitro* tests performed by Lee et al^103^ on CX1, LS174T, and HCT116 human colon cancer cells revealed that 15 mg/kg artesunate could dramatically reduce the expression of DR5 (death receptor 5), Bax (Bcl-2-associated X protein), and Bak (Bcl-2 homologous antagonist killer) as well as suppress tumor development. The process is associated with the up-regulation of DR5 and the improvement of ferroptotic cell susceptibility to TRAIL (tumor necrosis factor-related apoptosis-inducing ligand)-induced apoptosis.^103^ Related research has demonstrated that artesunate can control the degree of ferroptosis in tumor cells such as ovarian cancer cells, ovarian serous carcinoma cells, and Burkitt’s lymphoma cells.^104^, ^105^, ^106^

Dihydroartemisinin, which is produced from artemisinin by reducing sodium tetrahydroboronate, has the benefits of being more water soluble, having a fast metabolism, being quickly absorbed, being widely available, and having good anti-malarial action.^107^ By binding to cell-free iron, dihydroartemisinin can promote the binding of iron regulatory proteins to messenger RNA molecules that contain iron-responsive element sequences. As a result, it can interfere with the iron homeostasis process that is regulated by iron regulatory proteins and iron response elements.^108^ According to Du et al,^109^ dihydroartemisinin accelerates ferritin degradation, increases the unstable iron pool, encourages the formation of ROS in cells, and ultimately causes ferroptosis in acute myeloid leukemia cells by controlling the activity of the AMPK/mTOR (mammalian target of rapamycin)/p70S6k signaling pathway.^109^

### Cucurbitacin B

Cucurbitacin B, one of the most prevalent and extensively investigated cucurbitacin derivatives, is isolated from *Trichosanthes kirilowii Maximowicz*.^110^ It has proven potent anti-bacterial, anti-fungal, anti-pyretic, and anti-cancer properties that are applied in conventional medicine by controlling numerous signaling pathways.^111^, ^112^, ^113^ Huang et al^114^ showed that cucurbitacin B causes cell death via the ferroptosis pathway. The precise mechanism involves cucurbitacin B stimulation of the buildup of iron ions and the depletion of GSH levels, which leads to the overabundance of lipid peroxides. Additionally, cucurbitacin B inhibits GPX4 expression, which initiates a multifaceted ferroptosis pathway in CNE1 cells.^114^

### Deoxyelephantopin

Deoxyelephantopin is a plentiful sesquiterpene lactone that was discovered in *Elephantopus scaber*, a well-known perennial herb that has been used traditionally to cure hepatitis, infection, and diuresis.^115^^,^^116^ Chang et al^117^ discovered that deoxyelephantopin and its derivative DETD-35 are a novel GPX4 enzyme inhibitor that exerts its effect through noncovalent binding. By altering GSH levels and primary metabolism, lipid/oxylipin metabolism, and mitochondrial damage, deoxyelephantopin and DETD-35 caused lipid ROS buildup and triggered ferroptotic cell death in PLX4032-sensitive (A375) and PLX4032-resistant (A375-R) BRAF V600E melanoma cells.^117^

## Organic acids

Organic acids are a class of acidic organic chemicals that are commonly found in plants and include carboxyl groups. They are widely distributed in the leaves, roots, and notably the fruits of Chinese herbal medicine, where they primarily manifest as salts, fats, lipids, waxes, *etc*. Some of these substances have a role in the metabolism of animals and plants, while others are metabolic intermediates, exhibit important biological functions, or serve as raw materials for the pharmaceutical industry.

### Oleanolic acid

Oleanolic acid is a pentacyclic triterpene that is found naturally in plant leaves, fruits, and seeds.^118^ Numerous biological actions, including anti-oxidant, anti-inflammatory, and anti-tumor actions, have been linked to oleanolic acid.^119^, ^120^, ^121^ HeLa cells were used as the cellular model and tumor-bearing mice as the animal model in a study by Jiang et al.^122^ Oleanolic acid increased the levels of oxidative stress and Fe^2+^ content as well as the expression of proteins involved in ferroptosis in both *in vivo* and *in vitro* experiments. ACSL4 was also significantly up-regulated in cervical cancer (HeLa) cells and xenograft models after oleanolic acid therapy. Following the administration of siRNA to reduce ACSL4 expression in cervical cancer cells, the inhibitory effect of oleanolic acid on cell survival and proliferative capability was reversed, and a decrease in ROS levels and GPX4 was found. This finding implies that oleanolic acid decreases the survival rate of HeLa cells by promoting ACSL4 expression and activating ferroptosis in HeLa cells.^122^

### Gambogenic acid

Gambogenic acid, an active constituent of gamboge, a gum-resin extract of *Garcinia hanburryi* tree, is a TCM compound with excellent anti-cancer properties and regulatory effects on inflammatory and apoptosis pathways.^123^^,^^124^ Wang et al^125^ discovered that gambogenic acid greatly reduced the invasion, migration, and epithelial–mesenchymal transition of melanoma cells. These cells also had minor mitochondrial wrinkling, which is a crucial component of ferroptosis. Gambogenic acid increased the expression of the enzymes p53, GPX4, and SLC7A11, which contribute to the processes underlying gambogenic acid-induced ferroptosis. Moreover, their research points to the possibility that gambogenic acid triggers ferroptosis in TGF-1 (transforming growth factor 1)-stimulated melanoma cells through signaling via the p53/SLC7A11/GPX4 pathway.^125^

### Glycyrrhetinic acid

Glycyrrhetinic acid, also known as 18-β-glycyrrhetinic acid, is one of the primary active ingredients that has been identified from liquorice. Numerous pharmacological actions of glycyrrhetinic acid include anti-inflammatory, anti-tumor, anti-viral, and anti-oxidative properties.^126^, ^127^, ^128^, ^129^ Wen et al^130^ examined cell viability, apoptosis, and ferroptosis in MDA-MB-231 cells to demonstrate the anti-cancer effects of glycyrrhetinic acid. In MDA-MB-231 cells, glycyrrhetinic acid treatment boosted the expression and activity of NADPH oxidase and iNOS (inducible nitric oxide synthase) as well as the formation of ROS/reactive nitrogen species. Meanwhile, glycyrrhetinic acid reduced GSH levels, inhibited GPX function and down-regulated the expression of SLC7A11. Together, NADPH oxidases, iNOS, and decreased GSH and GPX activity might help glycyrrhetinic acid increase the generation of ROS and reactive nitrogen species, which then exacerbates lipid peroxidation and causes ferroptosis in triple-negative breast cancer cells.^130^

## Alkaloids

Alkaloids are a group of naturally occurring chemical compounds that are extensively present in plants, including those belonging to the families *Berberidaceae*, *Lycoraceae*, *Thyraceae*, and *Solanaceae*. All alkaloids include at least one basic nitrogen atom inserted into their ring structure. The biological effects of alkaloids are diverse, and in recent years, research on their anti-tumor properties has grown significantly.

### Solasonine

Solasonine, a steroidal alkaloid, is derived from *Solanum nigrum* L., a traditional Chinese herb. Numerous anti-tumor properties of solasonine have been discovered, including those that protect against lung cancer, bile duct cancer, and bladder cancer.^131^, ^132^, ^133^ Solasonine’s effects were studied *in vitro* and *in vivo* using pancreatic cancer cells (PANC-1 and CFPAC-1). Solasonine participates in ferroptosis by inhibiting the transcriptional up-regulation of OTUB1 (OTU deubiquitinase, ubiquitin aldehyde binding 1) mediated by TFAP2A (transcription factor AP-2 alpha), which in turn activates the ubiquitination-mediated degradation of SLC7A11 and encourages ferroptosis in pancreatic cancer cells. In summary, solasonine inhibits the TFAP2A/OTUB1/SLC7A11 axis to stimulate ferroptosis and prevent the spread of pancreatic cancer cells.^134^

### Berberine

Berberine is derived from the root of the natural plant *Coptis chinensis*. Alkaloids are extracted from the stem and have therapeutic properties, including anti-oxidative stress, hypoglycemic, and neuroprotective properties.^135^, ^136^, ^137^ In mice treated with berberine, the characteristic morphological alterations in ferroptotic cells and indicators of ferroptosis were mitigated, along with decreased malondialdehyde and ROS levels and increased GSH levels. In mice with cerebral ischemia‒reperfusion injury, berberine changed the composition of the gut flora, and antibiotics interfered with the protective effects of berberine. Based on the 16S rRNA data, KEGG analysis showed that berberine affected several metabolic pathways, including those involved in ferroptosis and GSH metabolism. This study raises the possibility that berberine can influence these pathways by modulating the microbiome to counteract ferroptosis caused by cerebral ischemia‒reperfusion injury and thereby demonstrate neuroprotective effects.^138^

### Leonurine

Leonurine, also named SCM-198, is extracted from the leaves of Chinese motherwort and has been reported to be protective against various cardiovascular and brain diseases.^139^^,^^140^ Leonurine hydrochloride can be used as a quality marker for motherwort identification and content estimation. In cisplatin-induced acute kidney damage, leonurine hydrochloride activated the Nrf2 antioxidative pathway, therefore protecting against alterations in ferroptosis-related morphological and biochemical markers, including malondialdehyde levels, superoxide dismutase and GSH depletion, and GPX4 and xCT down-regulation. However, in Nrf2 knockout mice, the protective effects of leonurine hydrochloride on acute kidney damage and ferroptosis were virtually eliminated. These findings indicate that lipid peroxide-mediated ferroptosis is at least partially inhibited to accomplish the renoprotective effects of Nrf2 activation on cisplatin-induced acute kidney damage.^141^ Similarly, a study demonstrated leonurine’s hepatoprotective efficacy against iron-induced hepatotoxicity, which may be achieved by altering Nrf2 and NF-κB signaling.^142^

## Others

There are structural types with fewer member compounds in addition to the above categories, and these structures cannot be classified using the categories indicated above.

### Bicyclol

*Schisandra chinensis*, a TCM, is the source of bicyclol, an innovative product pioneered in China. Bicyclol has several therapeutic benefits, including the prevention of viral replication, the reduction in oxidative stress, the prevention of fibrosis, the prevention of liver damage, and the promotion of protein synthesis in hepatocytes.^143^^,^^144^ In a study by Zhao et al,^145^ CCL4 (C–C motif chemokine ligand 4) induced iron accumulation, excessive production of ROS, increased lipid peroxidation, and distinctive morphological changes in mitochondria. Additionally, it resulted in a decrease in the levels of the proteins GPX4 and xCT in the livers of mice with acute liver injury, all of which are signs of ferroptosis. By favorably modulating the Nrf2/GPx4 axis to halt the aforementioned ferroptotic process, bicyclol protects the liver.^145^

### Astragalus polysaccharide

Astragalus polysaccharide (APS) is a natural bioactive substance found in *Astragalus membranaceus*, the root of *Astragalus membranaceus (Fisch.) Bge.*, or *Astragalus membranaceus (Fisch.) Bge. var. mongholicus (Bge.) Hsiao*.^146^ Its potential pharmaceutical effects include anti-cancer effects, radiation protection, immune system regulation, and gut microbiota modulation.^147^, ^148^, ^149^, ^150^ Chen et al^151^ found that astragalus polysaccharide treatment clearly inhibited ferroptosis in both dextran sulfate sodium-challenged mice and RSL3 (RAS-selective lethal 3)-stimulated Caco-2 cells, as demonstrated by a reduction in the expression of genes associated with ferroptosis (prostaglandin-endoperoxide synthase 2, ferritin light chain, and ferritin heavy chain) and the levels of surrogate ferroptosis markers (malondialdehyde, GSH, and iron accumulation). Their research revealed a novel function for astragalus polysaccharide in blocking the NRF2/HO1 pathway, which prevents ferroptosis in a murine model of experimental colitis and in human Caco-2 cells.^151^

### β-Elemene

β-Elemene is a powerful substance derived from the natural medicinal plant *Zedoary turmeric.* It is a tiny molecule and anti-tumor phytochemical medication discovered in China.^152^ Studies have shown that β-elemene can inhibit tumor growth by inducing apoptosis, limiting cell invasion and metastasis, and preventing new angiogenesis while also enhancing the function of the immune system.^153^^,^^154^ In KRAS mutant colorectal cancer cells, Chen et al^155^ showed that β-elemene and cetuximab caused iron-dependent ROS accumulation, GSH depletion, lipid peroxidation, up-regulation of HO1 and transferrin expression, and down-regulation of negative ferroptosis regulatory protein expression (GPX4, SLC7A11, FTH1, glutaminase, and SLC40A1). The effect was eliminated by ferroptosis inhibitors but not by other cell death suppressors. The results indicated for the first time that the natural substance β-elemene is a novel inducer of ferroptosis.

**Figure 3:**
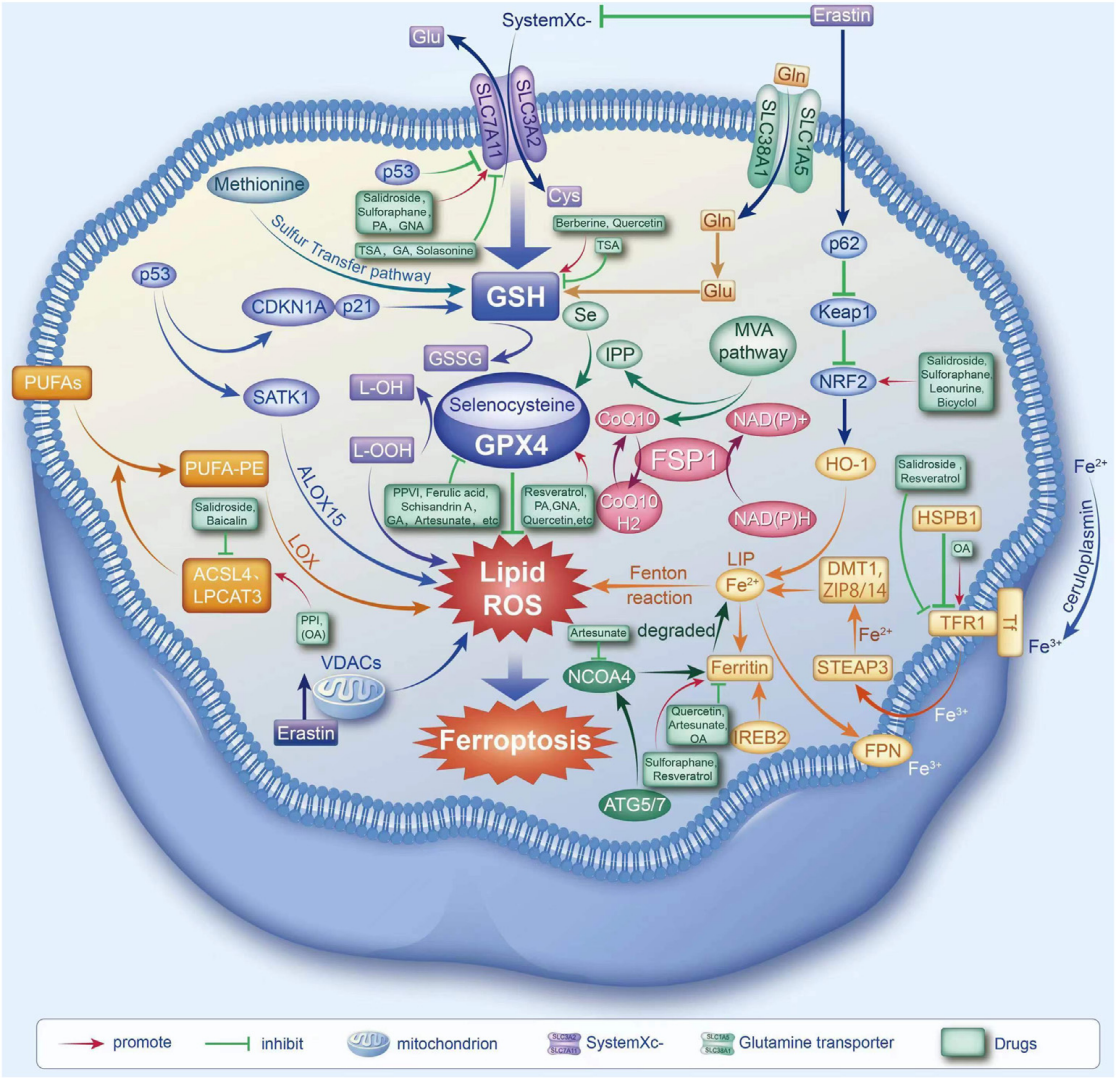
Significant traditional Chinese medicine (TCM) monomers involved in ferroptosis and their targets. OA, oleanolic acid; GA, glycyrrhetinic acid; PA, proanthocyanidin; GNA, gambogenic acid; TSA, tanshinone IIA.

To enhance comprehension, a schematic diagram illustrating the regulatory mechanism of ferroptosis by TCM monomers was created (Fig. 3).

## Prospects

Historically, novel medications have come largely from natural sources. Youyou Tu, a Nobel laureate, was inspired by the Zhouhou Beiji Fang, a well-known monograph of TCM written by Hong Ge in the Eastern Jin dynasty, to create artemisinin, which was received as a beneficial new therapy worldwide. More than 40 representative Chinese medicine compounds (Table 1) and monomers (Table 2) that modulate ferroptosis are outlined in this review, along with their targets and modes of action. Based on the relationship between the structure and efficacy of most natural drugs, drugs of the same pharmacological action type that bind to a specific receptor often have some structural similarity. Therefore, we categorized the compounds according to their structural properties to increase the medicinal plant resources and to find novel bioactive chemicals (Table 3).

**Table 1:** TCM compounds regulating ferroptosis.

TCM compounds	Composition	Function	Disease	Mechanisms
Fuzheng Kang’ai decoction	Radix Pseudostellariae, Rhizoma Atractylodis Macrocephalae, Milkvetch Root, etc.	Inducer	Non-Small Cell Lung Cancer	Augment lipid peroxidation and abundance of intracellular Fe2+ ions, downregulated expression of GPX4^13^
Qing-rehuo-xue formula	Scutellaria baicalensis and Radix paeoniae rubra	Inducer	Non-Small Cell Lung Cancer	Regulate the p53 and GSK-3β/Nrf2 signalling pathways^14^
Fuzheng Nizeng Decoction	Astragalus mongholicus Bunge, Astragalus mongholicus Bunge, Glycyrrhiza uralensis Fisch, etc.	Inducer	Gastric precancerous lesions	Downregulate GPX4/GSH levels; activation of ATF3/CHOP/CHAC1 signalling pathway^16^
Shuganning injection	Ganoderma Lucidum, Isatidis Radix, Gardeniae Fructus, Artemisiae Scopariae Herba, and a flavone glycoside baicalin	Inducer	Breast cancer	Upregulate the expression of HO-1/Nrf2^19^
DiDang Decoction	Rhubarb, Peach seed, Leech, and Gadfly	Inhibitor	Atherosclerosis and Hyperlipidaemia	Activate the HIF-1 signalling pathway^22^
Qing-Xin-Jie-Yu Granule	Astragalus membranaceu, Salvia miltiorrhiza Bunge, Ligusticum striatum, etc.	Inhibitor	Atherosclerotic cardiovascular diseases	Inhibit GPX4/xCT signalling pathway^24^
Fo-Shou-San decoction	Angelica sinensis and Ligusticum wallichii		Cognitive deficits	Modulate of the NRF2/HO-1 pathway^25^
Compound Tongluo Decoction	Radix Polygonum Multiflorum Preparata, the rhizome of Polygonatum sibiricum, the stem and leaf of seaweed, etc.	Inhibitor	Cerebral infarction	Upregulate the expression of GPX4, downregulate the expression of ACSL4^28^
Naotaifang	Radix Astragali, Rhizoma chuanxiong, Pheretima, and Bombyx batryticatus.	Inhibitor	Acute cerebral ischaemia	Modulate the TFR1/DMT1 and SCL7A11/GPX4 pathways^33^
QiShenYiQi dripping pills	Astragalus membranaceus Fisch. ex Bunge, Salvia miltiorrhiza Bge., Panax notoginseng (Burk.) F. H, etc.	Inhibitor	Myocardial ischaemia	Enhance mitochondrial biogenesis and dynamic homeostasis^35^
Niujiaodihuang Detoxify Decoction	Cornu Bubali, Paeonia lactiflora Pall, Rehmannia glutinosa, etc.	Inhibitor	Liver injury	ReprogramGSH metabolism^36^
Wenqingyin	Coptidis Rhizoma, Phellodendri Cortex, Gardeniae Fructus, etc.	Inhibitor	Liver injury	Activate the Nrf2-mediated signalling pathway^37^
Qingyi decoction	Bupleurum chinense DC, Corydalis yanhusuo, Scutellaria baicalensis Georgi, etc.	Inhibitor	Acute lung injury	Enhance ALDH2 expression and downregulate ICAM-1 expression^39^
Xiaojianzhong decoction	Cassia twig, Paeonia lactiflora, licorice, ginger, jujube, and maltose	Inhibitor	Gastric mucosal injury	Modulate the p62/Keap1/Nrf2 signalling pathway^40^

The Pharmacopoeia of the People’s Republic of China (version 2020) provided the names of these TCMs in both English and Chinese.

**Table 2:** Representative monomers of TCMs regulating ferroptosis.

TCMs or active pharmaceutical ingredients	Herbal Chinese name	Concentration	Function	Cell Types	Possible targets or mechanisms
PPIII	Chong lou	5 μM	Inducer	MDA-MB231 breast-carcinoma cells	ACSL4-mediated lipid peroxidation elevation^42^
PPⅥ	Chong lou	4 μM	Inducer	HCCLM3 and Huh7 cells	Inhibited the STAT3/GPX4 axis^43^
Salidroside	Hong jing tian	40 μM	Inhibitor	HT22 cells	Activated of the Nrf2/HO1 signalling pathway activated the Nrf2/GPX4 axis^44^
Sulforaphane	Gan lan	10 μM	Inhibitor	Neonatal mouse ventricular cardiac cells	AMPK-mediated NRF2 activation^48^
Ferulic acid	Dang gui	100 mg/kg	Inhibitor	Rat myocardial cells	Reduced ROS overproduction^52^
Psoralidin	Bu gu zhi	5.21 μM	Inhibitor	HT22 cells	Binds to 5-LOX, and the interface of Nrf2-Keap1 interaction^54^
Schisandrin A	Wu wei zi	50 μM	Inhibitor	Human renal glomerular endothelial cells	Activated AdipoR1/AMPK-ROS/mitochondrial damage^59^
Resveratrol	Li lu	10 μM	Inhibitor	H9c2 cells	Downregulated of USP19/Beclin1-induced autophagy^64^
Tanshinone IIA	Dan shen	250 μM	Inducer	SGC-7901 and BGC-823 cells	Increased the level of lipid peroxides and decreased GSH level^70^
Chrysophanol	Da huang	7.5 μM	Inducer	FaDu and SAS HNSCC cells	Lowered levels of GPX4 and lipid ROS^73^
Baicalin	Huang qin	25 μM	Inhibitor	H9c2 cells	Suppressed ACSL4 overexpression^81^
Proanthocyanidins	Hong hua	20 mg/kg/day	Inhibitor	Alveolar epithelial cells	Modulates the TGF-β1/Smad2/3 pathway^87^
Cyanidin-3-glucoside	Sang shen	50 μM	Inhibitor	HK-2 and NRK-52E cells	Activated the AMPK pathway^88^
Quercetin	Huai mi	50 μM	Inhibitor	INS-1 cells	Upregulated the levels of GSH and GPX4^94^
Artesunate	Huang hua hao	15 mg/kg	Inducer	CX1, LS174T, and HCT116 cells	Improved TRAIL-induced apoptosis sensitivity of ferroptotic cells^103^
Dihydroartemisinin	Huang hua hao	10 μM	Inducer	HL60, KG-1 and THP-1 cells	Modulated the AMPK/mTOR/p70S6k signalling pathway^109^
Cucurbitacin B	Gua lou	50 nM	Inducer	CNE1 cells	Encouraged the buildup of iron ions and the depletion of GSH, as well as inhibited GPX4 expression^114^
Deoxyelephantopin	Di dan cao	2.5 μM	Inducer	A-375 cells	Upregulated the levels of ROS^117^
Oleanolic acid	Nv zhen zi	5 μM	Inducer	HeLa cells	Promoted ACSL4 expression^122^
Gambogenic acid	Teng huang	1 μM	Inducer	A-375 and A-2058 cells	Modulated the p53/SLC7A11/GPX4 pathway^125^
Glycyrrhetinic acid	Gan cao	40 μM	Inducer	MDA-MB-231 cells	Induced ROS/RNS production, and increased lipid peroxidation.^130^
Solasonine	Long kui	50 μM	Inducer	PANC-1 and CFPAC-1 cells	Inhibited the TFAP2A/OTUB1 SLC7A11 axis^134^
Berberine	Huang lian	100 mg/kg	Inhibitor	Gut microbiota	Modulated the microbiome^138^
Leonurine	Yi mu cao	100 μM	Inhibitor	HK-2 cells	Partially inhibited lipid peroxide^141^
Bicyclol	Wu wei zi	10 μM	Inhibitor	L-02 cells	Positively regulated Nrf2-GPx4 axis^145^
Astragalus polysaccharide	Huang qi	50 μg/ml	Inhibitor	Caco-2 cells	Blocked the NRF2/HO-1 pathway^151^
β-elemene	E shu	125 μg/ml	Inducer	HCT-116 and LoVo cells	Upregulated the levels of ROS^155^

The Pharmacopoeia of the People’s Republic of China (version 2020) provided the names of these TCMs in both English and Chinese.

**Table 3:** Representative TCM monomers grouped based on molecular structural characteristics.

Species	Molecular structure characteristics	TCM monomers
Glycosides	An end-group carbon atom from a sugar or sugar derivative attached to a different type of non-sugar material to generate a compound	PPIII^42^; PPVI^43^; Salidroside^44^; SFN^48^; Astragaloside IV^158^; Timosaponin AIII^159^; Ophiopogonin B^160^; Saikosaponin A^161^; α-Hederin^162^; curculigoside^163^; Ophiopogonin D^164^; Paeoniflorin^165^; polyphyllin Ⅱ^166^
Phenylpropanoids	A family of naturally occurring compounds with three straight carbon chains (C6–C3) connecting the benzene ring	FA^52^; PSO^54^; Schisandrin A^59^; Esculetin^167^; Osthole^168^
Quinoid	Any member of the group of chemical compounds that have either the cyclohexadienedione or cyclohexadienedimethylene structure	Res^64^; TSA^70^; CHR^73^; Cryptotanshinone^169^; Dihydroisotanshinone I^170^; 15, 16-Dihydrotanshinone I^171^; Shikonin^172^; Acetylshikonin^173^
Flavonoids	The basic parent nucleus is 2-phenylchromogen	Baicalin^81^; PAs^87^; C3G^88^; QCT^94^; Icariside II^174^; Vitexin^175^; Chrysin^176^; Dihydromyricetin^177^; Galangin^178^; Baicalein^179^; Soybean isoflavones^180^; Calycosin^181^; Curcumin^182^; Ginkgetin^183^; Eriocitrin^184^; Isoorientin^185^; luteolin^186^
Terpenoids	Their fundamental structural unit is the isoprene unit.	ART^103^; DHA^109^; CuB^114^; DET^117^; Rehmannioside A^187^; β-caryophyllene^188^; Curcumenol^189^; β-Carotene^190^; β-Elemenic Acid^191^; Celastrol^192^; Obacunone^193^
Organic acids	Certain organic substances possessing acidic qualities	OA^122^; GNA^125^; GA^130^; Crocetin^194^; Ganoderic acid A^195^
Alkaloids	Alkaloids are nitrogen-containing organic molecules.	Solasonine^134^; BBR^138^; Leonurine^141^; Sinapine^196^; Sanguinarine^197^; Tetrandrine^198^; Matrine^199^
Others	These constructions cannot be categorized using the previously listed categories.	Bicyclol^145^; APS^151^; β-Elemene^155^; Bufotalin^200^; Cinobufotalin^201^; Neutral polysaccharide from Gastrodia elata^202^; Brusatol^203^; Erianin^204^; Andrographolide^205^

The Pharmacopoeia of the People’s Republic of China (version 2020) provided the names of these TCMs in both English and Chinese.

Before their functions in controlling ferroptosis had been investigated, the anti-oxidant properties of 11 of the 27 monomeric compounds were proposed. In Cisd2-deficient cardiomyocytes, ferulic acid substantially reduced oxidative stress-induced damage by enhancing catalase activity and by reducing endogenous ROS generation, as other researchers have shown in the past. According to a recent study, ferulic acid ameliorates oxidative stress, reduces ROS overproduction, promotes GSH production, and improves ischemia–reperfusion-induced ferroptosis. Vitamin K, also known as a natural vitamin with anti-oxidant effects, has recently been demonstrated to act as an anti-oxidant and to successfully inhibit ferroptosis.^156^ Since the defensive mechanisms against ferroptosis rely on cellular anti-oxidant systems to directly neutralize lipid peroxides, we can study a substance’s role and method in preventing ferroptosis considering its previously known anti-oxidant properties and get twice as much done with half the effort.

Natural products are a significant source of medicines since they exhibit original and varied structural features as well as distinctive biological activities.^157^ Natural medicines, nevertheless, can come with many drawbacks. For instance, due to their effective antimalarial properties and high absorption, artemisinin derivatives such as artemether and artesunate have displaced artemisinin as the most widely used anti-malarial medications in clinical practice. The structures of natural active compounds often need to be adjusted and improved since they might not be able to match the conditions necessary for drug production. Different approaches to chemical disposal are used depending on the molecular size and complexity of natural products. By analyzing the link between structure and activity, it is possible to extract pharmacophores, attain skeleton migration, and acquire novel molecular structures. Further, complex and large molecules can be structurally divided to remove unnecessary atoms, such as removing an extra chiral center while maintaining the structure and conformation required for binding to the target. Complete synthesis of a novel drug is often required for industrialization while safeguarding resources and the environment. This issue can be resolved using medicinal chemistry, resulting in new chemical entities with increased efficacy, drug resistance, and fewer harmful side effects, which will lower the cost of creating new leading compounds. The cycle of new medication research and development can be, in this way, accelerated.

We discovered that there was a significant variance in the ideal concentration of each monomer, even within the same molecular class. Both gambogenic acid and glycyrrhetinic acid belong to the class of organic acids, and the optimal concentrations are 1 μM and 40 μM, respectively. If we want to further investigate experimental pharmacology (animal studies) or clinical pharmacology (human studies), we must give priority to compounds with low concentrations. The incidence of pharmacological side effects or unpleasant responses rises dramatically due to a drug’s excessive concentration, which might harm the liver, kidneys, blood, and other systems. It is also noteworthy that different doses of the same substance have different pharmacological effects. As illustrated, tanshinone IIA (3–10 μM) was used to pretreat doxorubicin-damaged H9c2 cells and greatly reduced the production of ROS while raising the amount of intracellular GSH.^66^ By raising lipid peroxide levels and lowering GSH levels, both of which are signs of ferroptosis, tanshinone IIA (250 M) has the opposite impact on gastric cancer cells.^70^ The regulatory effects of the same substance on ferroptosis are completely opposite at different concentrations, and the specific reasons need to be further studied.

We found that several examples of monomers located in the original drug were also present in the compound. For example, *Wenqingyin* contains *Radix scutellariae radix* and *Angelica sinensis*, and *Qishen Yiqi Dripping Pill* contains *Radix astragalus membranaceus* and *Radix salvia miltiorrhiza*. The mechanisms of ferroptosis regulation by these monomers are different, and the mechanism changes when a different compound is formed. This phenomenon deserves further consideration. TCM is the result of real-world medical experience verified by five thousand years of practice. Many of the compounds have been repeatedly verified by countless people and in the treatment of countless diseases and have also been tested by more than 300 plagues from the Han dynasty to the Qing dynasty. However, even if the clinical effect is good, it is not widely recognized and applied in clinical practice due to the lack of sufficient evidence-based medicine. The pharmacological mechanisms of TCM compounds are mostly focused on a certain pathway, certain targets, or a certain type of functional activity in the treatment of diseases. With the help of these methods, researchers have partially revealed the mechanisms of multitarget drug action of TCM compounds at a certain level (the whole animal, organ, cell, or molecular level), but most of the studies are vague descriptions of the whole biological system before and after intervention or limited to a single target or a certain pathway and lack analysis at the system network level. In recent years, with the rise of new technologies such as systems biology and network pharmacology, the research and development of drug mechanisms and new drugs have been developed from the traditional research model of “single component, single target, single disease” to the direction of “multicomponent, multitarget, multipathway”. This provides a new idea for future research on the treatment of common diseases with similar multitarget TCM compounds.

## Summary

This paper presents a comprehensive examination of the correlation between ferroptosis and TCM in the context of disease treatment. This investigation establishes that the modulation of ferroptosis serves as a therapeutic avenue for TCM, thereby having significant implications for the utilization and advancement of TCM. Within this study, the mechanisms, chemical compositions, and optimal concentrations of Chinese medicine in regulating ferroptosis are thoroughly summarized and analyzed, thereby offering valuable insights for the development of targeted drugs aimed at manipulating ferroptosis.

## Funding

This study was supported by the 10.13039/501100001809National Natural Science Foundation of China (No. 82074171, 81700592).

## CRediT authorship contribution statement

**Shuai Liu:** Visualization, Writing – original draft. **Xianzhen Yang:** Writing – review & editing. **Sanxia Zheng:** Writing – review & editing. **Changjing Chen:** Writing – review & editing. **Lei Qi:** Visualization, Writing – review & editing. **Xiangdong Xu:** Conceptualization. **Denglu Zhang:** Conceptualization, Writing – original draft.

## Conflict of interests

The authors declared no conflict of interests.
